# Pyrimidyl formamidine palladium(II) complex as a nanocatalyst for aqueous Suzuki-Miyaura coupling

**DOI:** 10.1016/j.heliyon.2019.e01367

**Published:** 2019-03-20

**Authors:** Afaf Y. Khormi, Thoraya. A. Farghaly, Mohamed R. Shaaban

**Affiliations:** a Department of Chemistry, Faculty of Applied Science, Umm Al-Qura University, Makkah Almukaramah, Saudi Arabia; b Department of Chemistry, Faculty of Science, Cairo University, Giza 12613, Egypt

**Keywords:** Organic chemistry

## Abstract

Synthesis of a new phosphene-free nano-size formamidine-based palladium complex have been achieved. The molecular structure of novel palladium complex have been confirmed using spectroscopic methods of analysis as well as physical characterizations. The synthesized complex has been used as a catalyst for microwave assisted aqueous Suzuki-Miyaura Cross-coupling (SMC) of aryl bromides with phenylboronic acid. The formamidine-based Pd(II)-complex exhibited excellent catalytic activity to obtain biaryls using mild reaction conditions.

## 1 Introduction

Cross-coupling reactions with transition-metal-catalysts are considerably useful synthetic process for C-C bonds formation [1, 2, 3]. The Suzuki-Miyaura cross-coupling (SMC) [4], has become charming standard procedure for the synthesis of biaryls, which have an assorted spectrum of implementations, extending from pharmaceuticals to materials science [5]. Traditionally, palladium intermediates were stabilized by using phosphine ligands [6]. The air-susceptibility of these kinds of ligands, however, can prevent their use in a variety of synthetic implementations [7]. Moreover, phosphine ligands are often expensive and their price can override that of the palladium salt. On the other hand, phosphorus-free palladium catalysts have many advantages such as high air stability, thermal stability, higher catalytic activity which permit the catalyzed reaction to occur under mild reaction conditions [8, 9]. Thus, the development of ligands free from phosphine has been increasingly inspected in recent times, with amines, carbenes, thioureas, etc. [10, 11]. Construction of appropriate ligands might be a good controlling factor to obtain catalysts that push the weak leaving group such as chloride (in SMC) to take its role in the reaction and easily displaced. Also, developed appropriate ligands will led to catalysts that exhibit improved lifetimes, higher TON, recyclability and are stable to run the reactions in aerobic conditions at lower temperatures [12, 13]. Formamidines are multilateral ligands, qualified to form flexible coordination modes which display transition metal complexes with new electronic properties and recently showed excellent ability for stabilization of metals high oxidation states [14, 15, 16, 17, 18, 19, 20]. On the other side, it is predestined that main source of the reaction chemical waste is due to the solvent [21]. From green chemistry, safety and economic considerations, utility of water as a medium for chemical reactions is a target, although defy and in several cases demanding longer times and higher temperatures. In the case of the SMC, using aqueous medium causes the fastness of boronic acids which make utility of water as reaction medium a feature over other solvents [22]. Moreover, the use of microwave radiation as heating source in chemical reactions is a milestone in the development of green chemistry which reduces heat losses, increase the homogenous heating and reduce the by-products and consider as clean heat source [23, 24, 25]. However, numerous homogenous or heterogeneous palladium catalyst have been designed for microwave assisted Suzuki reactions [26, 27, 28, 29, 30, 31, 32, 33, 34], while, the formamidine palladium(II)-complexes utilized in catalysis of SMC are much little. Derived from the excellent results obtained in our previous works on the use transition metal complexes in various applications especially palladium(II)-complexes [35, 36, 37, 38, 39], we report here our study on the synthesis of new pyrimidyl formamidine-based palladium(II)-complex **4** (Fig. 2) and study its catalytic activity in the microwave assisted SMC of aryl bromides with phenylboronic acid under aerobic conditions in water.

## 2 Results and discussion

### 2.1 Synthesis and characterization of the Pd (II)-complex **4** (precatalyst **4**)

#### 2.1.1 Synthesis of the Pd (II)-complex **4** (precatalyst **4**)

**Fig. 1:**
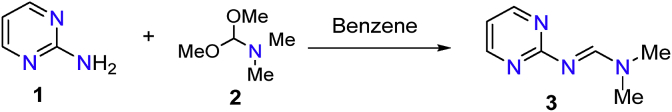
Preparation of the *N*, *N*-dimethyl-*N′*-(pyrimidin-2-yl) formimidamide **3**.

Reaction of 2-amino-pyrimidine (**1**) with dimethylformamide dimethylacetal (**2**) (DMF DMA), using benzene as a solvent according to previously reported procedure [40] afforded the corresponding *N*,*N*-dimethyl-*N'*-(pyrimidin-2-yl)formimidamide ligand **3** as depicted in Fig. 1. The structure of ligand **3** was confirmed by its spectroscopic data. Thus, the ^1^H NMR spectrum of the ligand **3** showed two singlet signals at *δ* 3.01 and 3.11 ppm due to the two methyl groups of *N*,*N*-dimethylamino group protons, in addition to a triplet at *δ* 6.91 ppm with coupling constant *J* = 4.8 Hz due to the C_5_-H pyrimidine ring proton, a doublet signal at *δ* 8.47 ppm with coupling constant *J* = 4.8 Hz due to the C_4_-H and C_6_-H pyrimidine ring protons and a singlet signal at *δ* 8.60 ppm due to the formamidine moiety proton. In addition, the infra-red spectrum of the ligand **3** showed the C=N group characteristic band at 1634 cm^−1^.

**Fig. 2:**
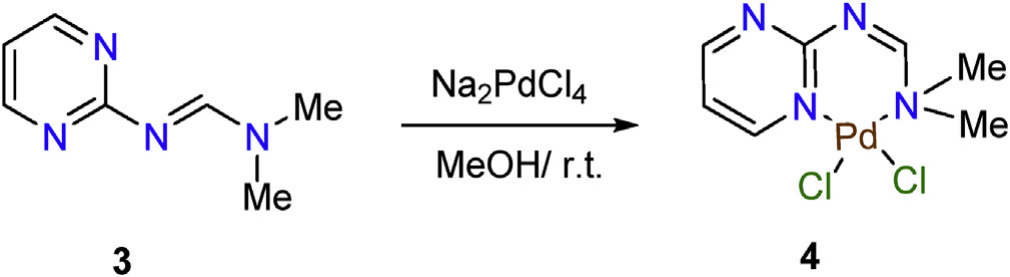
Synthesis of palladium complex **4** (precatalyst **4**).

The formamidine-based palladium(II) complex **4** was prepared by dissolving *N*,*N*-dimethyl-*N'*-(pyrimidin-2-yl)formimidamide ligand **3** in MeOH with consequent dropwise addition of equivalent moles of Na_2_PdCl_4_ (dissolved in methanol), at room temperature as depicted in Fig. 2. The molecular structure of the formamidine-based palladium(II) complex **4** was confirmed on the basis of its spectroscopic data as well as physical characterizations. Examining the ^1^H NMR spectrum of the formamidine-based palladium(II) complex **4** indicates the presence of a singlet at *δ* 3.28 ppm due to protons of N-Me_2_ group, in addition to multiplet signal at *δ* 7.0–7.20 ppm due to C_5_-H pyrimidine ring proton, a doublet signal at *δ* 8.50 ppm with coupling constant *J* = 5.4 Hz due to the C_4_-H, a doublet signal at *δ* 8.60 ppm with coupling constant *J* = 5.1 Hz due to C_6_-H pyrimidine ring protons and a singlet at *δ* 8.78 ppm due to the proton of formamidine N=CH group. Nitrogen atom coordination of the NMe_2_ group of formamidine moiety with the Pd affected the value of chemical shift (*δ)* of the protons of the two methyl groups of NMe_2_ group as well as N=CH formamidine proton, Thus, by comparison of the value of the chemical shift *δ* of the same group of protons in the ligand **3**, it was found that protons resonance occurs at down field region of the spectrum due to the electronic nature of Pd atom. In addition, IR spectrum of the formamidine-based palladium(II) complex **4** showed a vibrational band at 1624 cm^−1^ due to the presence of the C=N functional group and vibrational bands at 430 and 526 cm^−1^ due to the presence of Pd-N bond.

#### 2.1.2 Scanning electron microscopy (SEM) and EDX of precatalyst **4**

**Fig. 3:**
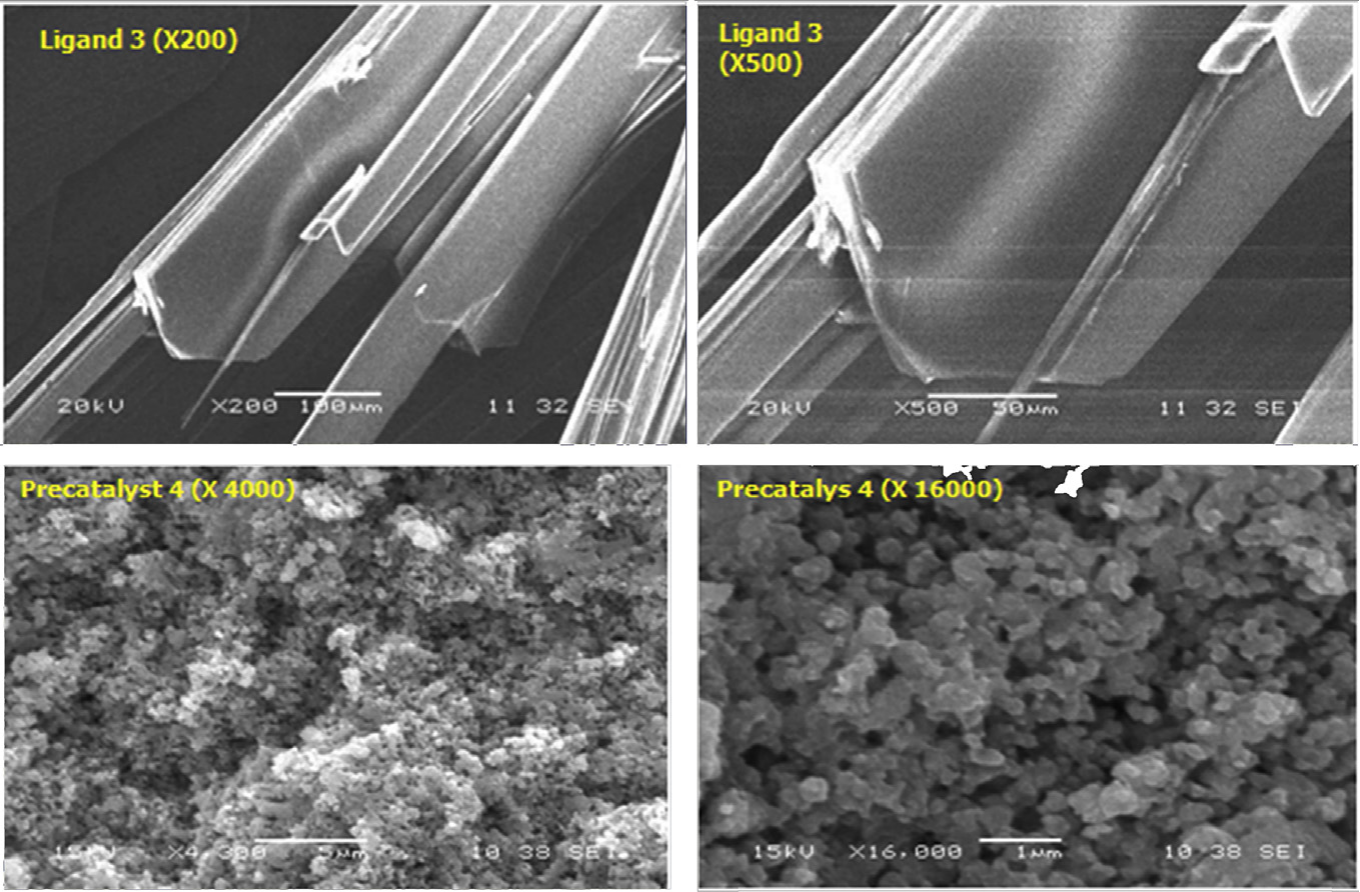
The SEM images of ligand 3 and precatalyst **4** with different magnifications.

**Fig. 4:**
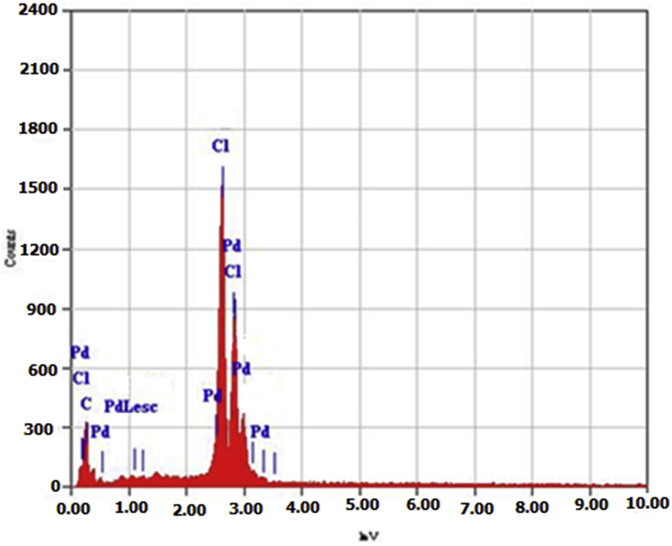
The EDX study of the precatalyst **4**.

The SEM images of catalyst **4** and ligand **3** with different magnifications are presented in Fig. 3. As it can be seen, different morphologies of the catalyst are observed. The particles are agglomerated in different shapes and sizes compared with the ligand **3**. Also, the spherical shape of the catalyst particles encourages their catalytic activity. The EDX study of the catalyst (Fig. 4) reveals that, the catalyst surface composed of Pd, Cl and carbon atoms with different concentrations.

#### 2.1.3 X-ray diffraction (XRD) analysis of precatalyst **4**

**Fig. 5:**
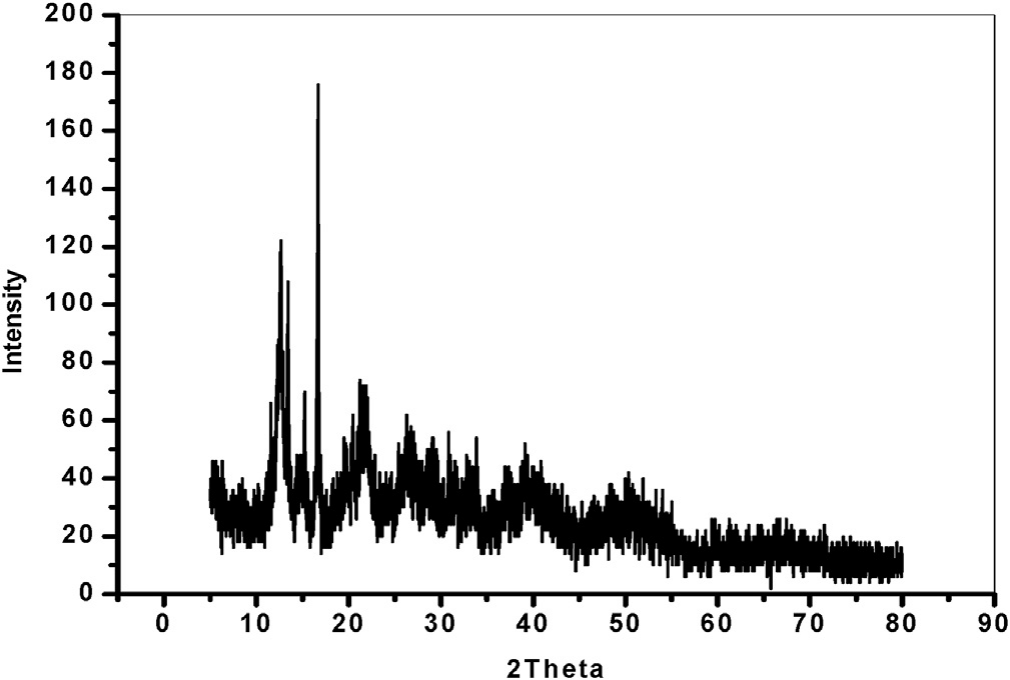
X-ray diffraction (XRD) analysis of precatalyst **4**.

The crystalline structure of the prepared formamidine-based palladium(II) complex **4** was examined and characterized by X-ray diffraction (XRD) analysis, the results are presented in Fig. 5. Careful inspection of Fig. 5 reveals that, the catalyst has well crystalline diffraction peaks at 2*θ* between 5° to 30°. Moreover, metallic palladium (2*θ* = 39.9^o^, 46.2^o^ and 67.8^o^) (JCPDS-ICDD Card No. 46-1043) diffraction peaks can be identified [41]. The formation of Pd nanoparticles is originated during the catalyst preparation procedures. Also, no peaks characteristic to palladium oxide can be observed in the XRD analysis of the formamidine-based palladium(II) complex **4**. The XRD results confirm the formation of metallic Pd nanoparticles which agree well with SEM images results.

Thus, Deby–Scherrer equation:

B = 0.94 *λ*/(S Cos *θ*)

S is the crystallite size.*θ* is the diffraction angle.B is the line width at half maximum height, Cu/K*α* (*λ*) = 1.5406 A°.

The above equation was used to calculate the crystallite size, where the calculated size is present excellently in nanometer range which equal 8.763 nm.

#### 2.1.4 Thermogravimetric analysis (TGA) of formamidine-based palladium(II) complex **4**

**Fig. 6:**
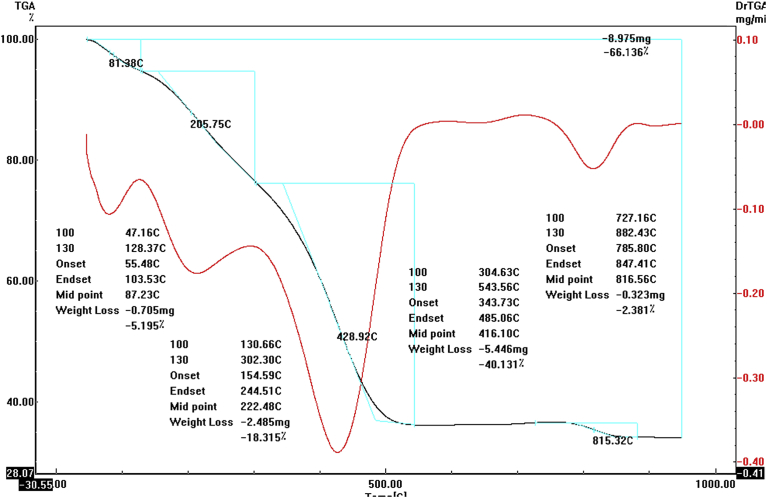
Thermogravimetric analysis (TGA) of precatalyst **4**.

Fig. 6 shows thermogravimetric analysis of (1:1) complex which formed between *N*,*N*-dimethyl-*N'*-(pyrimidin-2-yl)formimidamide ligand **3** and sodium tetrachloropalladate. The obtained results of TGA are listed in Table 1. As shown in the thermogram, the weight loss percent for the 1^st^ step in the temperature range 47–131 °C was corresponding to the loss of one hygroscopic water molecule with weight loss 5.37% (Calc. 5.21). Whereas the second thermal degradation step in temperature range of 131–310 °C was attributed to the loss of two chloride ions with weight loss 18.9% (Calc. 21.3). The weight loss in the temperature range 310–891 °C was a result of the decomposition of organic part of complex. The final product is palladium oxide which has weight loss of 66.1% (theoretically 64.6%).

**Table 1:** Results of thermogravimetric analysis of (1:1) (Pd:formamidine-based ligand).

Complex chemical formula (M.wt)	Step order	Temperature range (°C)	Process	Weight loss %	
				Calc.	Found
[PdPymCl_2_] H_2_O (345.4)	1^st^	47–131	1- Loss of a water molecule	5.21	5.37
[PdPymCl_2_] (327.4)	2^nd^	131–310	2- Loss of two chloride ions	21.3	18.9
[PdPym] (256.4)	3^rd^	310–891	3- Loss of organic part and PdO formation	64.6	66.1

### 2.2 Catalytic study

#### 2.2.1 Optimization and factors affecting the catalytic ability of formamidine-based palladium(II) complex **4** in SMC

##### 2.2.1.1 Effect of catalyst concentration

**Fig. 7:**
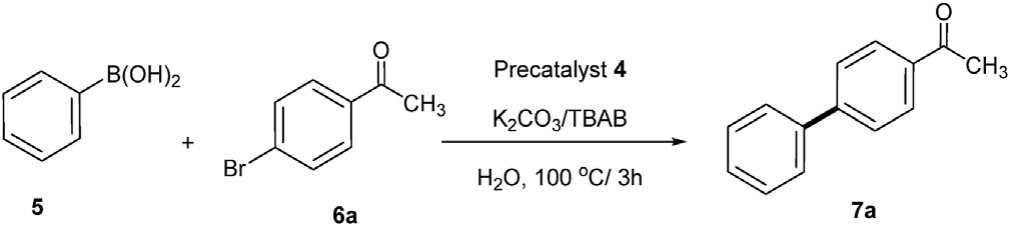
Effect of concentration of precatalyst **4** on the coupling of 4′-bromoacetophenone (**6a**) with phenylboronic acid (**5**) in water.

In order to judge the catalytic activity of the developed catalyst we have started by the study of the effect of concentration of precatalyst 4 on the C-C cross-coupling of phenylboronic acid (5) with 4′-bromoacetophenone (6a) (model reaction), using water as a solvent, in the presence of potassium carbonate as a base and a co-catalyst ((tetrabutylammonium bromide) (TBAB)). Thus, under thermal reaction conditions heating the reaction mixture in the presence of different concentrations of the precatalyst 4 for three hours at 100 °C afforded the corresponding cross coupling product, 4-acetyl-1,1′-biphenyl (7a) as shown in Fig. 7 and Table 2. In the beginning, the reaction was carried out using one mol% of the precatalyst 4 with a molar ratio of 6a: 5: K_2_CO_3_: TBAB is 1:1.2:1:0.6, to give 100% conversion (based on GC-analysis) of the cross coupling product 7a (entry 1, Table 2). In entries 2-5, 0.75–0.125 mol% of the precatalyst 4 were used which gave also full GC-conversion under the same reaction conditions (Table 2). When PdCl_2_ is used in the presence of ligand 3 instead of precatalyst 4 we obtained lower conversion of the cross-coupling (entry 7, Table 2). From the data in Table 2, it can be concluded, that the precatalyst 4 showed excellent catalytic ability in the SMC even at very low concentrations. Expectedly, the starting 4′-bromoacetophenone (6a) was completely recovered when the reaction was carried out without the precatalyst 4 (entry 7, Table 2). The structure of the obtained product, 4-acetyl-1,1′-biphenyl (7a) was confirmed by spectroscopic data (see experimental part).

**Table 2:** Effect of concentration of precatalyst **4** on the yield, TON, and TOF of the coupling of 4′-bromoacetophenone (**6a**) with phenylboronic acid (**5**) in water.

Entry	Pd, mol%	Conversion %^a^^,^^b^	TON	TOF (h^−1^)
1	1.00	100	100	33
2	0.75	100 (95)	133	44
3	0.50	100	200	66
4	0.25	100	400	133
5	0.125	100	800	266
6	0.05	89	1780	593
7	1.00 (PdCl_2_/ligand 3)^c^	87	87	26
8	0.00	0	0	0

a Conditions: 4′-bromoacetophenone (6a)/phenylboronic acid (5)/base/TBAB/water: 1/1.2/1/0.6/10 mL, under thermal heating at 100 °C for 3 h.

b Conversions were based on GC-analysis and the value between parenthesis refer to the highest isolated yield%.

c Instead of precatalyst 4 1.00 mol% of PdCl_2_ in the presence of 1.00 mol% of ligand 3 was used.

In order to clarify the catalytic role of the precatalyst in the cross coupling, it should be noted that the palladium complex serves like “dormant species” that does not actually participate in the reaction catalytic cycle but it is considered as a main source of a catalytically active species related to palladium of unknown nature. In general, the Pd(0) species was reported in many literature to be the true active catalysts. Therefore, the precatalyst 4 may serve in this catalytic cycle as a reservoir that is indirectly involved in cross coupling but is main source of release of nano-sized Pd(0) which can show catalytic activity even at low concentrations [42]. Attempts for reusability of the precatalyst have been done using the same experimental conditions, unfortunately we could not get reasonable results even with using the higher mol% of the precatalyst 4.

##### 2.2.1.2 Effect of solvent and base on SMC using precatalyst 4 under thermal conditions

**Fig. 8:**
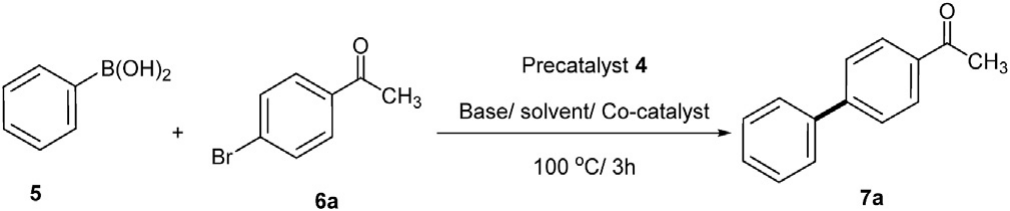
Effect of base and solvent Suzuki cross coupling of 4′-bromoacetophenone (**6a**) with phenylboronic acid (**5**) thermally.

The catalytic efficiency of the precatalyst 4 have been examined in order to get full conversions of the reactants and high products yields. Thus, different parameters that may affect such C-C cross-coupling have been optimized. The reaction media and type of base are among the controlling factors that affect the yield optimization. In general, there is no rule for selection of the base and the choice of it is still empirical, thus, the combination between different bases and a variety of solvents for the coupling reaction between 6a and phenylboronic acid (5) were investigated. As presented in Fig. 8 and Table 3, in all cases, the cross coupling was carried out using different media *e.g.* water, dimethylformamide (DMF), toluene, benzene, acetonitrile (AN), dioxane and tetrahydrofuran (THF) using 0.75 mol% concentration of the precatalyst 4 and in the presence of appropriate base such as potassium hydroxide (KOH), potassium carbonate (K_2_CO_3_) or triethylamine (TEA) under thermal conditions. The optimization also included the use of different types of co-catalyst such as tetrabutylammonium bromide (TBAB), tetrabutylammonium iodide (TBAI), tetrabutylammonium tetrafluoroborate (TBATFB), cetyl dimethyl benzyl ammonium bromide (CDBAB), cetyl trimethyl ammonium bromide (CTAB). The best results were obtained with water solvent using K_2_CO_3_ as a base in the presence of variety of the co-catalyst after refluxing at 100 °C (entries 1-4, Table 3). Also, in many cases we achieved 100% conversions by using nonpolar organic solvents as shown in Table 3 (entries 8, 10, and 15). When water was replaced with DMF, acetonitrile, dioxane, and THF in the presence of K_2_CO_3_, the reaction conversions were 77, 84, 100, and 88%, respectively (entries 6,7,9, and 11, Table 3). While the replacement of K_2_CO_3_ with KOH as a base improved the conversions when DMF and THF were reaction solvent, it is not suitable in case of water as a solvent (entries 13, 14, and 16, Table 3). Toluene was proved to be suitable solvent for the cross coupling regardless the type of the base used (entries 8, and 15, Table 3). Finally, Also TEA as an organic base using water afforded 83% conversion (entry 12, Table 3).

**Table 3:** Effect of base and solvent Suzuki cross coupling of 4′-bromoacetophenone (**6a**) with phenylboronic acid (**5**) under thermal conditions.

Entry	Base	Solvent/co-catalyst	Yield%^a^
1	K_2_CO_3_	H_2_O/TBAB	100 (95)
2	K_2_CO_3_	H_2_O/TBAI	100 (90)
3	K_2_CO_3_	H_2_O/TBATFB	100 (92)
4	K_2_CO_3_	H_2_O/CDBAB	100 (90)
5	K_2_CO_3_	H_2_O/CTAB	80 (78)
6	K_2_CO_3_	DMF	77 (75)
7	K_2_CO_3_	Acetonitrile	84 (83)
8	K_2_CO_3_	Toluene	100 (83)
9	K_2_CO_3_	Dioxane	100 (95)
10	K_2_CO_3_	Benzene	84 (80)
11	K_2_CO_3_	THF	88 (87)
12	TEA	H_2_O/TBAB	83 (80)
13	KOH	H_2_O/TBAB	90 (87)
14	KOH	DMF	100 (93)
15	KOH	Toluene	100 (91)
16	KOH	THF	100 (81)

a Conversion by GC-analysis and the value between parenthesis indicates the product isolated yield%. Conditions: 4′-Bromoacetophenone/phenylboronic acid/co-catalyst (if used)/base/solvents: 1/1.2/0.6/1/10 mL, under thermal heating at 100 °C for 3 h.

The decision of choice of a solvent for palladium-catalysts is of the most important controlling parameters, specifically its complexing properties. From environmental point of view, H_2_O as an eco-friendly solvent and K_2_CO_3_ as a cheap base and considered as the common base for carrying out all the SMC of aryl bromides in the presence of TBAB as a co catalyst that were used as optimized conditions here this work.

#### 2.2.2 SMC using precatalyst **4** under microwave irradiation (MW)

**Fig. 9:**
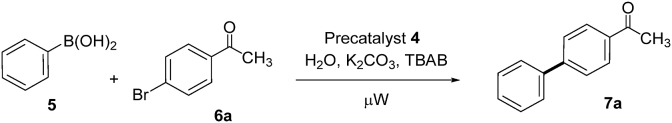
Cross-coupling of 4′-bromoacetophenone (**6a**) with phenylboronic acid (**5**) under microwaves conditions.

Using the optimized conditions, the model cross-coupling reaction in water was carried out under microwave conditions (MW) at different MW power as shown in Fig. 9 and Table 4. The cross coupling reaction 6a and phenylboronic acid (5) was carried out using the precatalyst 4 (0.75 mol%) with a molar ratio of 6a/5/TBAB/K_2_CO_3_: 1/1.2/0.6/1 to give full conversion and 98% yield of 4-acetyl-1,1′-biphenyl (7a) after only 10 min of microwaves irradiation.

**Table 4:** Cross-coupling of 4′-bromoacetophenone (**6a**) with phenylboronic acid (**5**) under microwaves conditions using different conditions.

Entry	Temp. (°C)	Time (min)	μW power (W)	Conversion %^a^
1	100	10	100	92
2	95	10	100	92
3	95	10	200	96
4	95	10	300	97
5	95	12	300	97
6	90	10	300	85
7	95	10	300	100(98)^b^
8	95	10	400	100
9	95	10	500	100

a Conditions: p-bromoacetophenone/phenylboronic acid/TBAB/K_2_CO_3_/water: 1/1.2/0.6/1/10 mL.

b Isolated yield.

#### 2.2.3 SMC of different aryl bromides with phenylboronic acid using precatalyst **4** under microwaves irradiation conditions

**Fig. 10:**
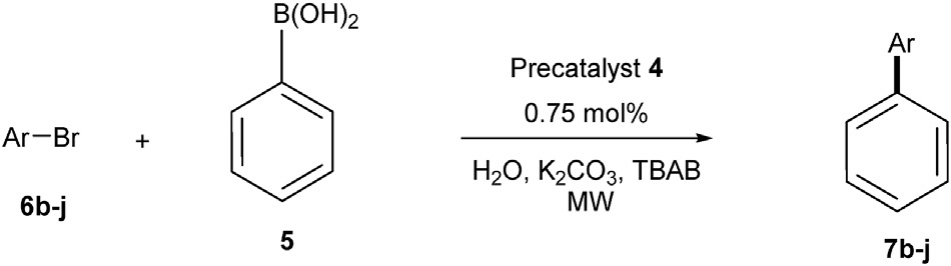
Suzuki cross-coupling of aryl bromides **6b-j** with phenylboronic acid (**5**) using the prcatalyst **4** under microwaves conditions.

Applying the optimized conditions (water/K_2_CO_3_/TBAB) under microwaves irradiation, SMC between aryl bromides 6b-j and phenylboronic acid (5), using 0.75 mol% of the precatalyst 4, resulted in the corresponding biaryl derivatives in moderate to excellent yields as shown in Fig. 10 and Table 5. Cross coupling of 1-bromonaphthalene 6i with the phenylboronic acid (5) afforded the corresponding 1-phenyl naphthalene in lower yield than the other aryl bromides (Table 5). The obtained results showed the reasonable catalytic efficiency of the precatalyst 4 towards Suzuki cross-coupling of aryl bromides 6b-j with phenylboronic acid (5).

**Table 5:** Yield % of Suzuki cross-coupling of aryl bromides **6b-j** with phenylboronic acid (**5**) using the prcatalyst **4** under microwaves conditions.

Compd. No.	Ar	Isolated yield%
6b, 7b	-C_6_H_4_-2-COCH_3_	80
6c, 7c	-C_6_H_4_-4-OCH_3_	84
6d, 7d	-C_6_H_4_-2-OH	61
6e, 7e	-C_6_H_4_-4-OH	98
6f, 7f	-C_6_H_4_-4-CF_3_	60
6g, 7g	-C_6_H_4_-2-CHO	80
6h, 7h	-C_6_H_4_-4-CHO	96
6i, 7i	-1-Naphyl	40
6j, 7j	-6-Naphyl-2-OH	92

Conditions: Aryl bromide/phenylboronic acid/TBAB/K_2_CO_3_/water: 1/1.2/0.6/1/10 mL, microwave heating (300 W) at 95 °C for 10 min.

## 3 Experimental

All melting points were measured on a Gallenkamp melting point apparatus. The infrared spectra were recorded in potassium bromide discs on a Pye Unicam SP 3–300 and Shimadzu FT IR 8101 PC infrared spectrophotometers. The NMR spectra were recorded in dimethyl sulfoxide (DMSO-*d*_*6*_) or CDCl_3_.on a Varian Mercury VXR-300 NMR spectrometer. Chemical shifts were related to that of the solvent. Mass spectra were recorded on a Shimadzu GCMS-QP1000 EX mass spectrometer at 70 eV. The Microwave irradiation was carried out on a CEM mars machine. CEM has several vessel types that are designed for their ovens: closed-system vessels including the HP-500 (500 psig material design pressure and 260 °C), have liners are composed of PFA and are ideal for many types of samples. HP-500 Plus vessels are ideal for routine digestion applications. Process up to 14 high-pressure vessels per run with temperatures up to 260 °C or pressures up to 500 psi.

### 3.1 Synthesis

#### 3.1.1 Preparation of *N*,*N*-dimethyl-*N'*-(pyrimidin-2-yl)formimidamide 3

A mixture of 2-aminopyrimidine (1) (5 mmol) and DMF-DMA (2) (10 mmol) in benzene (30 mL) was refluxed for 6 hours. After the reaction was complete, the mixture was cooled in room temperature and the white product of low melting was separated. Purification of the product have been achieved by flash column chromatography using chloroform as eluent to give 3 (75 % yield). IR (KBr) 1634, 1585, 1570, 1397, 1283 cm^−1^. ^1^H NMR (CDCl_3_) *δ* 3.01 (s, 3H, CH_3_), 3.11 (s, 3H, CH_3_), 6.91 (t, 1H, *J* = 4.8 Hz, C_5_-H pyrimidine), 8.47 (d, 2H, *J* = 4.8 Hz, C_4_-H and C_6_-H pyrimidine), 8.60 (s, 1H, CH-formamidine); MS *m/z* (%) 150 (M^+^).

#### 3.1.2 Synthesis of the palladium (II)-complex **4**

A methanolic solution of sodium tetrachloropalladate (1 mmol) was added dropwise to a stirred solution of the formamidine 3 (1 mmol) in methanol (10 mL). After stirring for 1 h at room temperature, the green precipitate was filtered off, washed with methanol and dried. The complex 4 was obtained as green powder (80%). Mp. 239 °C; IR (KBr) 1624, 1550, 1534, 1490, 1386, 1302, 819, 526, 430 cm^−1^. ^1^H NMR (DMSO-*d*_*6*_) *δ* 3.28 (s, 6H, 2-CH_3_), 7.0–7.20 (m, 1H, C_5_-H pyrimidine), 8.50 (d, 1H, *J* = 5.4 Hz, C_4_-H pyrimidine), 8.60 (d, 2H, *J* = 5.1 Hz, C_6_-H pyrimidine), 8.78 (s, 1H, CH-formamidine).

#### 3.1.3 Suzuki coupling of aryl bromides

##### 3.1.3.1 Effect of concentration of the Pd-complex 4 on the Suzuki coupling of 4-bromoacetophenone (6a) with phenylboronic acid (5) in water under thermal conditions

A mixture of 4′-bromoacetophenone (6a) (199 mg, 1 mmol) and phenylboronic acid (5) (146 mg, 1.2 mmol), tetrabutylammonium bromide (TBAB) (194 mg, 0.6 mmol), palladium complex 4 (4.03 mg, 1 mol%), K_2_CO_3_ (138 mg, 1 mmol) and water (10 mL) was refluxed for 3 h at 100 °C to give 4-acetyl-1,1′-biphenyl (7a). The same experiment was repeated using palladium complex 4 in (0.75, 0.5, 0.25, 0.125, 0.05 and 0.00) mol%. The molar ratio of the reaction components were in all cases as follows; 4-bromoacetophenone, phenylboronic acid, TBAB, K_2_CO_3_, water: 1/1.2/0.6/1/10 mL water. The yield% versus concentration of palladium complex 4 is outlined in Table 2.

**4-Acetyl-1,1′-biphenyl (7a):** Colorless solid; mp. 118–120 °C (Lit. [43] mp. 119–120 °C). ^1^H NMR (CDCl_3_): *δ* = 2.65 (s, 3H, COCH_3_), 7.27–7.51 (m, 3H), 7.63–7.65 (d, 2H), 7.68–7.71 (d, 2H), 8.03–8.06 (d, 2H). MS (EI, 70 eV): *m/z* (%) = 196 [M^+^].

##### 3.1.3.2 Effect of base and solvent on Suzuki cross-coupling of 4-bromoacetophenone (6a) with phenylboronic acid (5) under thermal heating

A mixture of 4-bromoacetophenone (6a) (199 mg, 1 mmol) and phenylboronic acid (5) (146 mg, 1.2 mmol), TBAB (194 mg, 0.6 mmol) (in case of using water as a solvent), Pd-complex 4 (0.75 mol%), a base (1 mmol) and solvent (10 mL) was stirred under reflux in open air for 3 hour to give acetyl-1,1′-biphenyl (7a). The molar ratio of the reaction components were, in all cases, as follows; 4-bromoacetophenone, phenylboronic acid, tetrabutylammonium bromide (in case of water), base, solvent: 1/1.2/0.6/1/10 ml. The yield % versus different solvents and bases is outlined in Table 3.

##### 3.1.3.3 Suzuki cross-coupling of aryl bromides with phenylboronic acid in water under microwave irradiation

###### 3.1.3.3.1 General procedure

A mixture of the appropriate aryl bromides 6b-j (1 mmol), and phenylboronic acid (5) (146 mg, 1.2 mmol), tetrabutylammonium bromide (194 mg, 0.6 mmol), Pd-complex 4 (0.75 mol %), K_2_CO_3_ (138 mg, 1 mmol), and distilled water (10 mL) were mixed in the specified CEM reaction vessel HP-500. The mixture was heated under microwave irradiating conditions at 95 °C and 300 Watt for 10 min. After the reaction was complete (monitored by TLC), the reaction mixture was extracted with ethyl acetate (3 × 20 mL). The combined organic extracts were dried over anhydrous Na_2_SO_4_ then filtered and the solvent was evaporated under reduced pressure. The products 7b-j were purified by flash column chromatography as described above. The yields% are outlined in Table 5.

**2-Acetyl-1,1′-biphenyl (7b):** mp. 54–57 °C (lit [44] mp. 54–57 °C); ^1^H NMR (CDCl_3_) δ 2.65 (s, 3H, COCH_3_); 7.37–7.66 (m, 7H); 7.83 (d, 1H); 8.5 (d, 1H). MS (EI, 70 eV): *m/z* (%) = 196 [M^+^].

**4-Methoxy-1,1′-biphenyl (7c):** mp. 87–88 °C (lit [45] mp. 87–88 °C); ^1^H NMR (CDCl_3_) δ 3.87 (s, 3H, -OCH_3_), 6.99 (d, 2H), 7.27–7.45 (m, 3H), 7.54 (d, 2H), 8.3 (d, 2H).

**2-Hydroxy-1,1′-biphenyl (7d):** mp. 57–59 °C (lit. [46] mp. 59 °C); ^1^H NMR (CDCl_3_)δ 5.4 (s, 1H, OH), 7.02 (d, 1H), 7.26 (d, 1H), 7.40–7.43 (m, 6H), 7.50 (d, 1H). MS (EI, 70 eV): *m/z* (%) = 170 [M^+^].

**4-Hydroxy-1,1′-biphenyl (7e):** mp. 164–166 °C (lit. [47] mp. 164-165); ^1^H NMR (CDCl_3_) δ 5.1 (s, 1H, OH), 6.91 (d, 2H), 7.27–7.58 (m, 7H). MS (EI, 70 eV): *m/z* (%) = 170 [M^+^].

**4-(trifluoromethyl)-1,1′-biphenyl (7f):** mp.70–72 °C (lit. [48] mp. 70–72 °C); ^1^H NMR (CDCl_3_) δ: 7.27–7.63 (m, 7H), 8.25 (d, 2H). MS (EI, 70 eV): *m/z* (%) = 222 [M^+^].

**2-phenyl benzaldehyde (7g):** Colorless oil lit [49]; ^1^H NMR (CDCl_3_) δ: 7.38–8.06 (m, 9H), 10.0 (s, 1H, CHO). MS (EI, 70 eV): *m/z* (%) = 182(49.3) [M^+^].

**4-phenyl benzaldehyde (7h):** mp. 57–59 °C (lit. [49] mp. 57–59 °C); ^1^H NMR (CDCl_3_) δ: 7.43–7.66 (m, 5H), 7.67 (d, 2H), 7.86 (d, 2H), 10.07 (s, 1H, CHO). MS (EI, 70 eV): *m/z* (%) = 182(49.3) [M^+^].

**1-Phenylnaphthalene (7i):** Colorless oil lit [50]; ^1^H NMR (CDCl_3_) δ 7.43–7.94 (m, 12H). MS (EI, 70 eV): *m/z* (%) = 204 [M^+^].

**6-Phenyl-2-naphthol (7j):** yellow oil lit [51]; ^1^H NMR (CDCl_3_) δ 5.3 (s, 1H, OH), 7.1 4–7.98 (m, 11H). MS (EI, 70 eV): *m/z* (%) = 220 [M^+^].

## 4 Conclusions

We developed a new formamidine-based palladium(II) complex as robust, stable efficient nano-sized catalyst for aqueous Suzuki-Miyaura cross-coupling of aryl bromides with phenylboronic acids under microwave irradiation. The pyrimidyl formamidine-based palladium complex showed a high catalytic activity, high even at low mol% concentrations, in mild reaction conditions.

## Declarations

### Author contribution statement

Mohamed R. Shaaban: Conceived and designed the experiments; Analyzed and interpreted the data; Contributed reagents, materials, analysis tools or data; Wrote the paper.

Thoraya. A. Farghaly: Conceived and designed the experiments; Analyzed and interpreted the data; Wrote the paper.

Afaf Y. Khormi: Performed the experiments; Analyzed and interpreted the data; Wrote the paper.

### Funding statement

This research did not receive any specific grant from funding agencies in the public, commercial, or not-for-profit sectors.

### Competing interest statement

The authors declare no conflict of interest.

### Additional information

No additional information is available for this paper.
